# The Clinical Role of Insulin Degludec/Insulin Aspart in Type 2 Diabetes: An Empirical Perspective from Experience in Australia

**DOI:** 10.3390/jcm9041091

**Published:** 2020-04-11

**Authors:** Sarah J. Glastras, Neale Cohen, Thomas Dover, Gary Kilov, Richard J. MacIsaac, Margaret McGill, Greg R. Fulcher

**Affiliations:** 1 Department of Diabetes, Endocrinology and Metabolism, The Royal North Shore Hospital, University of Sydney, Reserve Road, St Leonards NSW 2065, Australia; greg.fulcher@sydney.edu.au; 2 Baker Heart and Diabetes Institute, 75 Commercial Road, Melbourne VIC 3004, Australia; neale.cohen@baker.edu.au; 3 Ipswich Hospital, University of Queensland, Chelmsford Avenue, Ipswich QLD 4305, Australia; tomdover@hotmail.com; 4 Mater Hospital Brisbane, Raymond Terrace, South Brisbane QLD 4101, Australia; 5 Launceston Diabetes Clinic, 247 Wellington Street, Launceston TAS 7250, Australia; garykilov@gmail.com; 6 Department of General Practice and Primary Health Care, University of Melbourne, 230 Gratton Street, Parkville VIC 3010, Australia; 7 Department of Endocrinology & Diabetes, St Vincent’s Hospital Melbourne, University of Melbourne, 41 Victoria Parade, Fitzroy VIC 3065, Australia; r.macisaac@unimelb.edu.au; 8 Diabetes Centre, Royal Prince Alfred Hospital, Sydney NSW 2050, Australia; margaret.mcgill@sydney.edu.au

**Keywords:** insulin degludec/insulin aspart, IDegAsp, co-formulation, type 2 diabetes, Australia

## Abstract

Treatment intensification in people with type 2 diabetes following failure of basal insulin commonly involves the addition of a rapid-acting insulin analogue (basal plus one or more prandial doses; multiple daily injections) or by a switch to premixed insulin. Insulin degludec/insulin aspart (IDegAsp), comprising rapid-acting insulin aspart and ultra-long-acting insulin degludec in solution, enables both fasting and post-prandial glucose control, with some advantages over other treatment intensification options. These include straightforward dose titration, flexibility in dose timing, low injection burden, simplicity of switching and a lower risk of hypoglycaemia. In Australia, where insulin degludec on its own is not available, IDegAsp enables patients to still benefit from its ultra-long-acting properties. This review aims to provide guidance on where and how to use IDegAsp. Specifically, guidance is included on the initiation of IDegAsp in insulin-naïve patients, treatment intensification from basal insulin, switching from premixed or basal-bolus insulin to IDegAsp, up-titration from once- to twice-daily IDegAsp and the use of IDegAsp in special populations or situations.

## 1. Introduction

Diabetes affects over 422 million people globally and is associated with significant personal and societal burden [1]. Many individuals will require insulin therapy to control blood glucose levels due to the well-described progressive deterioration of beta cell function [1,2].

IDegAsp is a co-formulation of ultra-long-acting insulin degludec and fast-acting insulin aspart in solution [3]. As such, the individual pharmacokinetic properties of each insulin are retained, providing an advantage over other insulin mixtures. In addition, IDegAsp is currently the only option in Australia for prescribing insulin degludec; this has shaped the clinical use of IDegAsp in Australia and also in other countries. Therefore, it is considered a good management option for both insulin initiation and intensification of insulin therapy.

The following sections aim to provide guidance on where and how to use IDegAsp in real-world practice, in both Australian diabetes clinics and primary care.

### 1.1. Glycaemic Control in T2D

Chronic hyperglycaemia is associated with an increased risk of micro- and macro-vascular disease [4,5,6]; achieving tight glycaemic control involves balancing the risk of hypoglycaemia with optimal control of both fasting (FPG) and post-prandial glucose (PPG) levels [7,8]. Guidelines commonly recommend an individualised glycated haemoglobin (HbA1c) goal with a default target of <53 mmol/mol (<7.0%) for most insulin-requiring individuals with type 2 diabetes (T2D) [9,10], though more stringent goals (<48 mmol/mol; <6.5%) are recommended for some (younger patients with minimal comorbidities) and less stringent goals (~64 mmol/mol; ~8.0%) for others (elderly patients or those with hypoglycaemic unawareness) [9].

### 1.2. Pharmacological Management of T2D

Alongside lifestyle modification, oral antidiabetic drugs (OADs) with or without injectable glucagon-like peptide-1 receptor agonists (GLP-1 RAs) are the most usual treatment options for T2D, individualised according to the patient’s clinical status [9,11,12,13]. However, for many patients, insulin is eventually required to achieve glycaemic treatment goals [12,13,14]. A common approach is to initiate basal insulin with intermediate or long duration of action (e.g., isophane, insulin detemir, insulin glargine) [12].

Many patients treated with basal insulin alone eventually require intensification of insulin to reach post-prandial glycaemic targets [12,15]. Such an approach could include a basal plus rapid-acting insulin analogue (e.g., insulin aspart, insulin glulisine or insulin lispro) [12,13,14] or a tailored basal-bolus regimen. Basal-bolus therapy provides both basal and precise PPG control as separate injections, but is often viewed as complex, particularly when prandial doses are calculated according to mealtime carbohydrate estimates and because of the necessity for multiple injections, and potentially increasing fear of hypoglycaemia [12,16]. Despite this, basal-bolus therapy provides patients with flexibility enabled by variable prandial doses and timing of insulin injection.

**Figure 1:**
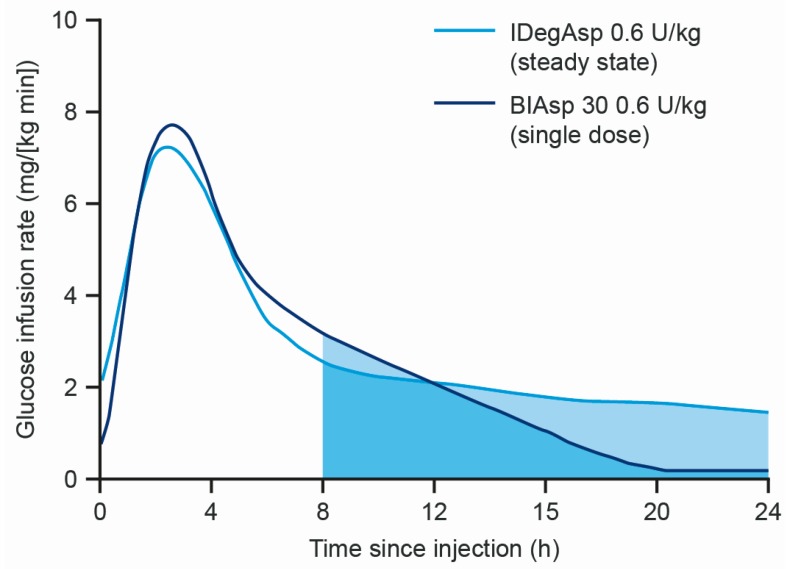
Comparison of the mean glucose infusion rate of IDegAsp and BIAsp30 in subjects with T1D [19]. BIAsp 30, biphasic insulin aspart 30/70; IDegAsp, insulin degludec/insulin aspart co-formulation; T1D, type 1 diabetes; U, units.

An alternative intensification option is the use of a premixed insulin, a suspension of protaminated insulin and rapid-acting insulin [17] in one device (e.g., biphasic insulin aspart 30/70 (BIAsp 30)), meaning fewer daily injections [12]. Drawbacks include lack of 24 h basal coverage (because of the intermediate duration of action of protaminated insulin); limited flexibility of dose timing (for the same reason) preferentially suiting patients with relatively fixed patterns of meal ingestion, in addition to the need for manual re-suspension of the insulins prior to injection; and the interaction between the short-acting and longer-acting insulin in suspension. This creates a ‘shoulder effect,’ whereby the duration of action of the short-acting insulin is prolonged after a meal, increasing the propensity for hypoglycaemia due to unwanted prolonged glucose-lowering action (Figure 1) [18,19].

In clinical practice, insulin initiation, titration and intensification are often delayed, even when glycaemic targets are clearly exceeded for a sustained period of time [20,21,22]. Reasons for clinical inertia include perceived complexity of managing multiple daily injections of insulin, fear of hypoglycaemia and a lack of adequate guidance to identify when treatment intensification is needed [22]. There is an unmet need for insulin products or regimens that are effective, well tolerated and simple to use in order to give patients, their caregivers and their treating clinicians the confidence to initiate and adjust insulin therapy as needed, and achieve optimal levels of glycaemic control.

### 1.3. The IDegAsp Co-Formulation

IDegAsp is the first fixed-ratio co-formulation of two different insulin analogues, comprising rapid-acting insulin aspart (30%) and ultra-long-acting insulin degludec (70%) in solution [23,24].

**Figure 2:**
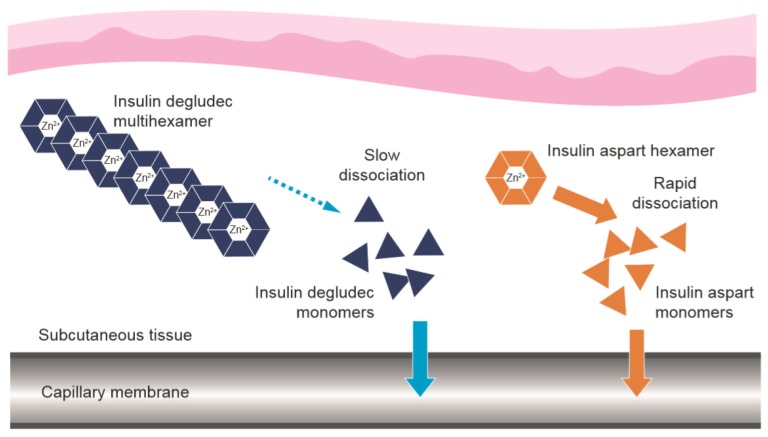
Dissociation of the components of IDegAsp following subcutaneous injection [27]. IDegAsp = insulin degludec/insulin aspart co-formulation. The use of Figure 2 from “Distinct Prandial and Basal Glucose-Lowering Effects of Insulin Degludec/Insulin Aspart (IDegAsp) at Steady State in Subjects with Type 1 Diabetes Mellitus” by Heise, T. et al. (2015) [18] is licensed under CC BY 4.0.

The basal component, insulin degludec, exhibits flat and stable steady-state pharmacokinetic and pharmacodynamic profiles in patients with diabetes [25,26]. Stable and soluble multihexamers are formed upon injection, from which insulin monomers slowly and gradually dissociate (Figure 2) [27], providing a duration of action >42 h with a half-life of approximately 25 h [28,29]. Insulin degludec achieves a steady-state in 2–3 days [30,31] and exhibits lower day-to-day variability in glucose-lowering effect compared to insulin glargine [32,33], with a relatively lower risk of hypoglycaemia [34,35,36]. Importantly, dose timing can be flexible from day to day (within 8–40 h of the previous dose with once-daily (OD) dosing) [37]. In countries where insulin degludec is available, these properties have made it a popular choice.

Following subcutaneous injection, the prandial component, insulin aspart, is rapidly absorbed following dissociation of the insulin hexamers into monomers (Figure 2) [27]. Insulin aspart is designed to provide a fast onset and short duration of glucose-lowering action [38,39], ideal for limiting post-prandial hyperglycaemia, with a low risk of late post-prandial hypoglycaemia.

The self-association and dissociation properties of insulin degludec and insulin aspart mean that the two molecules do not interact in the co-formulation or after injection, preserving their distinct pharmacological profiles [30,40]. Therefore, the rapid-acting analogue advantage at mealtimes is retained, combined with truly flat 24 h basal profile of insulin degludec, decreased variability after injection and no ‘shoulder effect’ after meals, as would be the case with premixed insulins. The IDegAsp co-formulation provides both FPG and PPG control, and allows flexibility in the timing of dosing (e.g., for people travelling across time zones, shift workers or those who rely on healthcare providers to administer their insulin during home visits) in a simple regimen with fewer injections than basal-bolus regimens and a lower rate of hypoglycaemia than premix, basal-plus or basal-bolus regimens [41,42,43]. Moreover, due to the co-formulation, there is no need to gently mix the insulins prior to injection.

### 1.4. Current Guidance on the Practical Use of IDegAsp

It is important that practical guidance/recommendations relevant to the treatment landscape in Australia, where insulin degludec is not available, are accessible to diabetes care providers. The following sections aim to provide guidance on where and how to use IDegAsp in the real world and in different clinical scenarios. The recommendations are based on clinical trial results, recommendations from current management guidelines, the published literature and discussions at an expert consensus meeting held in Sydney in 2018 involving many of the authors. Guidance is included on the initiation of IDegAsp in insulin-naïve patients, IDegAsp titration algorithms, treatment intensification from basal insulin to IDegAsp and from OD to twice daily (BID) IDegAsp, switching from premixed or basal-bolus insulin to IDegAsp and the use of IDegAsp in special populations or situations.

## 2. Clinical Guidance for the Use of IDegAsp in People with T2D

### 2.1. Initiation of IDegAsp in Insulin-Naïve People with T2D

**Figure 3:**
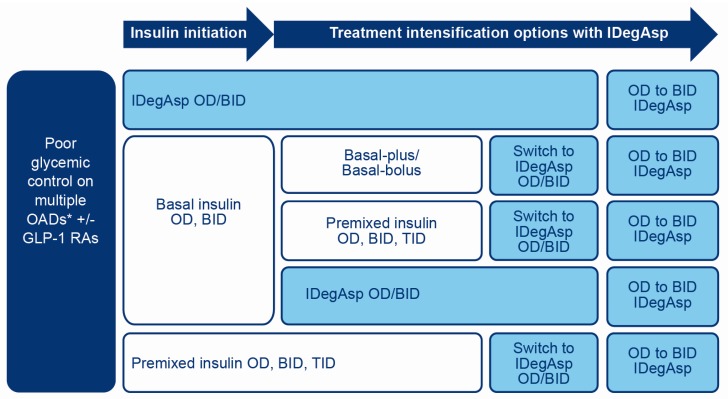
Situations where IDegAsp could be considered in T2D patients requiring insulin therapy. *Sulfonylurea, TZD, DPP-4i, SGLT2i. BID, twice daily; DPP-4i, dipeptidyl peptidase-4 inhibitor; GLP-1RA, glucagon-like peptide-1 receptor agonist; IDegAsp, insulin degludec/insulin aspart co-formulation; OAD, oral antidiabetic drug; OD, once daily; SGLT2i, sodium-glucose cotransporter-2 inhibitor; TID, three times daily; T2D, type 2 diabetes; TZD, thiazolidinedione.

IDegAsp may be considered for initiation of insulin in people with T2D following an inadequate response to OADs (Figure 3) and is preferable to basal insulin alone when prandial glucose control is required [42,44].

#### 2.1.1. Clinical Evidence of Efficacy

A number of studies support the use of IDegAsp for insulin initiation in people with T2D (Table 1). IDegAsp OD was superior to glargine U100 in reducing HbA1c levels in the BOOST JAPAN trial [45], and IDegAsp BID was non-inferior to BIAsp 30 BID (in terms of HbA1c control) in the START TWICE DAILY trial [46]. Although the absolute rates of hypoglycaemia observed during the studies were low, IDegAsp also resulted in lower rates of nocturnal and overall hypoglycaemia compared to glargine U100 [47] or BIAsp 30 [46].

#### 2.1.2. Practical Recommendations

The recommended total daily starting dose of IDegAsp is 10 units administered before the most carbohydrate-heavy meal of the day, and subsequent individual dosage weekly adjustments made until the desired FPG is reached [3]. Some clinicians may prefer to initiate IDegAsp once daily at a larger initial starting dose (particularly in a person with poor glycaemic control), or initiate BID dosing when unacceptable post-prandial hyperglycaemia is apparent at two or more mealtimes. In cases where PPG is still uncontrolled but FPG is satisfactory, insulin aspart may be added to the meal(s) where glycaemic control is unsatisfactory or patients may be advised to reduce carbohydrate intake at that meal. IDegAsp may be administered in combination with background therapies [23,43] but sulfonylureas should be discontinued [44], particularly when two injections of IDegAsp are given.

To initiate IDegAsp BID, the estimated total daily dose may be split into two equal or unequal doses, between the two largest meals, ensuring at least 4 h between each dose to avoid ‘stacking’ [30] of the short-acting insulin component [3,19,23]. ‘Stacking’ of insulin degludec does not occur.

IDegAsp is simple to titrate and two titration schemes were found to be effective in the Phase 3 clinical trial programme. In a simple algorithm, the IDegAsp dose was titrated using 2-unit increments or decrements (or no change if target achieved) based on a single pre-breakfast self-measured blood glucose (SMBG) measurement on the day of titration [48]. In a stepwise algorithm, doses of IDegAsp were titrated once weekly using increments or decrements of 2–8 units based on the lowest of three consecutive pre-breakfast SMBG readings (two days before, and on the day of titration) [48]. In our opinion, weekly titration is preferable in most real-life clinical situations, and titration shorter than three-day intervals should be avoided. In the clinic, adjustments to the total daily dose of IDegAsp should be based on FPG levels. When BID dosing is used, meal size and post-prandial increases should determine how the doses are split.

### 2.2. Insulin Intensification from Basal Insulin to IDegAsp

IDegAsp may be considered for treatment intensification in people with T2D with inadequate glycaemic control on basal insulin (Figure 3).

#### 2.2.1. Clinical Evidence of Efficacy

A number of studies support the use of IDegAsp for treatment intensification in people with T2D not attaining glycaemic targets on basal insulin (Table 1). IDegAsp OD gave similar glycaemic control to glargine U100 + insulin aspart in the Step-by-Step intensification study [43] and IDegAsp BID was superior to BIAsp 30 in the Intensify All trial [51]. Rates of nocturnal confirmed hypoglycaemia were also lower with IDegAsp in both studies [43,51].

#### 2.2.2. Practical Recommendations

Patients on basal insulin OD may switch unit-to-unit to IDegAsp OD, or from basal insulin BID to IDegAsp BID, at the same total daily insulin dose as previously [3,44]. However, if HbA1c is raised, then a clinician may elect to commence IDegAsp at a higher dose equivalent to the total daily insulin. Conversely, in some contexts, if HbA1c is not raised, or if patients are receiving high-dose basal insulin, many clinicians prefer to decrease the dose (by ~20%) when switching insulin to minimise risk of hypoglycaemia. Dose titration on a weekly basis is a sensible approach to then attain glycaemic targets. Patients on basal insulin OD may alternatively be started on IDegAsp BID by splitting the prior total dose into two equal or unequal doses of IDegAsp [51,54,55]. Monitoring of post-prandial glucose profiles prior to switching will likely inform as to whether a dose reduction is required and whether IDegAsp OD or IDegAsp BID would be the most appropriate option.

### 2.3. Intensification from IDegAsp OD to IDegAsp BID

If HbA1c is not at target in patients receiving IDegAsp OD, then more extensive glucose monitoring is needed to determine where hyperglycaemia is occurring. If there are post-prandial glucose excursions after two meals, which are unresponsive to diet manipulation, IDegAsp may be intensified from OD to BID (Figure 3). This is an alternative strategy to adding a rapid-acting insulin analogue such as insulin aspart alongside IDegAsp OD. It has the advantage of maintaining a flat basal insulin profile, maintaining a single insulin together with its device and avoiding a large dosage of insulin aspart delivered at a single meal. If there are three post-prandial glucose excursions, then it is advisable to intensify treatment from IDegAsp OD to IDegAsp BID and add a single dose of insulin aspart at the third meal.

#### 2.3.1. Clinical Evidence of Efficacy

Results from the Step-by-Step study confirmed that intensification from IDegAsp OD to IDegAsp BID can be safely implemented without negatively impacting glycaemic control (Table 1) [43]. IDegAsp OD was non-inferior to glargine U100 OD with insulin aspart OD with respect to HbA1c levels, and no differences in glycaemic parameters were seen between IDegAsp OD/BID and glargine U100 OD with insulin aspart OD/BID/thrice daily (TID) (Table 1) [43]. IDegAsp OD or IDegAsp OD/BID also resulted in lower rates of nocturnal hypoglycaemia compared to glargine U100 OD + IAsp OD or glargine U100 OD + IAsp OD/BID/TID, respectively [43]).

#### 2.3.2. Practical Recommendations

The total daily dose of IDegAsp OD may be split into two injections per day (BID), to be given at each of the two most carbohydrate-heavy meals. The ratio of split (not necessarily 50:50) should be based on the relative size of the meals with respect to carbohydrate content, the PPG excursion following each meal and the FPG level. The total basal dose should be adjusted according to FPG readings. While this adjustment in the trials was made every three days, in clinical practice a weekly dose adjustment is recommended, and the ratio of split calculated according to meal size and/or post-breakfast and dinner glucose increase. This is different to premix adjustment protocols: when titrating the dose of BIAsp 30, the lowest of the three previous days’ pre-meal blood glucose levels should be used (NovoMix 30 PI); the dose should not be increased if hypoglycaemia occurred within these days and dose adjustments are made once a week until target HbA1c is reached.

### 2.4. Switching from Premixed Insulin to IDegAsp

People with T2D not achieving adequate glycaemic control on premixed insulin may be switched to IDegAsp (Figure 3). This approach is straightforward, with the advantage of reducing delayed post-prandial hypoglycaemia, which is particularly concerning in patients taking premixed insulin with the evening meal who may be at risk of nocturnal hypoglycaemia.

#### 2.4.1. Clinical Evidence of Efficacy

In the Intensify Premix I trial, IDegAsp BID resulted in similar HbA1c levels and gave superior FPG control to BIAsp 30 BID in patients switching from premixed insulin (Table 1) [41]. Rates of hypoglycaemia were also significantly lower with IDegAsp. Similar results were seen in a pooled analysis of the Intensify Premix I and Intensify All trials (Table 1) [52].

#### 2.4.2. Practical Recommendations

IDegAsp should usually be started at the same unit dose and injection schedule as the premixed insulin [3]. As per the approved IDegAsp label, patients switching from premixed insulin OD can be converted unit-to-unit to IDegAsp OD, or from premixed insulin dosed BID or TID to IDegAsp BID, at the same total daily insulin dose [23,44]. In some cases, however, the starting dose of IDegAsp may be reduced by 10%–20% from the previous premixed dose requirement, according to clinician discretion [44]. People previously receiving premixed insulin once daily may alternatively be started on IDegAsp BID by splitting the prior total dose into two equal or unequal doses of IDegAsp [44,51,55]. Patients switching from a single morning injection of premix to IDegAsp BID should be carefully monitored for hypoglycaemia.

Patients frequently use rapid-acting insulin analogues at mealtimes in combination with their premixed insulin and should continue to use them at the same dose for meals not covered by IDegAsp [3]. IDegAsp should only be used either OD or BID dosing, and TID dosing should be avoided. Use of rapid-acting insulin analogues alongside IDegAsp allows additional dosing flexibility in some patients.

### 2.5. Switching from Basal-Plus or Basal-Bolus Insulin to IDegAsp

People with T2D not achieving adequate glycaemic control on basal-plus (basal insulin plus a single prandial insulin dose at the main meal) or basal-bolus insulin (basal insulin plus prandial insulin before each meal), or requiring simplification of a complex regimen, may be switched to IDegAsp (Figure 3).

#### 2.5.1. Clinical Evidence of Efficacy

In the Step-by-Step intensification study, IDegAsp OD was non-inferior to glargine U100 OD + insulin aspart OD with respect to HbA1c levels, and no difference was seen between IDegAsp OD/BID and glargine U100 OD + insulin aspart OD/BID/TID with respect to glycaemic parameters (Table 1) [43]. There were also significantly fewer nocturnal confirmed hypoglycaemic episodes with IDegAsp.

#### 2.5.2. Practical Recommendations

Switching from basal-plus or basal-bolus insulin therapy to IDegAsp requires individualisation according to the glycaemic profile. In general, however, following basal-plus therapy, IDegAsp may be given OD. Following basal-bolus therapy, IDegAsp should be started BID at the two main meals, initially at the same dosage as the basal insulin, split into two doses (not necessarily 50:50) and then titrated to achieve optimal FPG. A clinician may elect to omit a further dose of rapid-acting insulin to simplify the insulin regimen, or may choose to continue insulin aspart or its equivalent at the mealtime not covered by IDegAsp, at the same dose as previously or at a lower dose [3].

For those on basal-bolus regimens requiring four or five injections per day, some patients may be able to achieve adequate glycaemic control with IDegAsp BID; others may require an alternative approach using a three-dose intensive regimen (IDegAsp with the main carbohydrate meal and insulin aspart with the other two meals) [44]. Close glucose monitoring is recommended during the transfer and in the following weeks [23].

## 3. Use of IDegAsp in Special Populations or Situations

### 3.1. Elderly Patients

The elderly may be considered a good target group for treatment with IDegAsp; the simple and flexible dosing regimen will benefit those who have their insulin administered by visiting nurses or relatives as it can be administered at various times of the day in conjunction with the visit and the main meal. Elderly patients with reduced dexterity may struggle with regimens that involve a large number of daily injections, e.g., basal-bolus insulin. Available IDegAsp delivery systems are the RYZODEG^®^ 70/30 FlexTouch^®^ and the RYZODEG^®^ 70/30 Penfill^®^ (Table S1) [24,56]. The RYZODEG^®^ 70/30 FlexTouch^®^ device may be the best choice for elderly patients in terms of ease of use.

A combined post-hoc analysis of elderly patients with T2D in the Phase 3 Intensify Premix I and Intensify All trials showed that IDegAsp BID was efficacious with no need for special safety precautions [57]. In addition, the pharmacokinetic properties of IDeg are not affected by age [58]. Nevertheless, greater caution should be exercised when insulin is administered to elderly patients with diabetes as they may be more susceptible to hypoglycaemia [59,60]. The initial dosing, dose increments and maintenance dosage should be conservative and individualised (with less stringent HbA1c targets in many cases), and glucose monitoring intensified [3,23]. Taking a thorough diet history is essential before commencing IDegAsp, especially in older persons who may have significant variability in quantity and quality of carbohydrate intake.

### 3.2. Patients on a Very-Low-Calorie, Reduced-Carbohydrate or Erratic Diet

Variability in dietary patterns and practices (e.g., low carbohydrate, ketogenic diet or intermittent fasting), an erratic lifestyle with variability of meal choice and timing, illness or religious practices such as Ramadan can influence glycaemic control in people with diabetes [61]. IDegAsp may be useful in these situations due to flexibility in the dosing schedule [42], though its use should be cautioned in some instances, particularly if carbohydrate ingestion is limited at the meal with which IDegAsp is injected.

In a randomised treat-to-target trial, IDegAsp BID provided sustained glucose control before, during and after Ramadan fasting in patients with T2D previously treated with basal or premixed insulin ± OADs, with a significantly lower risk of hypoglycaemia than BIAsp 30 BID [62]. Doses of IDegAsp BID and BIAsp 30 BID were reduced by 30%–50% on the first day of Ramadan and readjusted to pre-Ramadan levels at the end of Ramadan.

### 3.3. Patients with Hepatic or Renal Impairment

Although the effect of hepatic or renal impairment on the pharmacokinetics of IDegAsp has not been studied directly [3], the effects of its components, insulin degludec and insulin aspart, have been studied. The pharmacokinetic profile of insulin degludec was found to be similar in patients with renal or hepatic impairment and individuals without impairment [63,64]. Similarly, the pharmacokinetics of insulin aspart are unaffected by renal or hepatic impairment [65]. These results suggest that dose adjustments of IDegAsp will not necessarily be required as a result of renal or hepatic impairment [63,64].

In the context of chronic illness, especially end-stage kidney disease or later stages of liver cirrhosis, appetite and body weight can deteriorate significantly. Close monitoring of glucose profiles can identify trends towards lower glucose levels and avoid significant hypoglycaemia in these vulnerable patient populations. IDegAsp can be used in renal or hepatic impaired patients with intensive glucose monitoring and the usual dose adjustments on an individual basis [3,23].

### 3.4. Hospitalised Patients

When initiating IDegAsp in hospital, clinicians should be aware of the long half-life of the insulin degludec component of this co-formulation. IDegAsp is not considered suitable for situations in which rapid inpatient glycaemic control is desired. Acutely unwell patients in hospital may need to stop using IDegAsp and be switched to a basal-bolus insulin regimen to prevent hyperglycaemia. IDegAsp may also not be the preferred choice for steroid-induced hyperglycaemia, especially when steroid doses are rapidly changing, given the long duration of the insulin degludec component.

### 3.5. Use in Pregnancy

There are no available data with IDegAsp or insulin degludec in pregnant women to inform a drug-associated risk for major birth defects and miscarriage. IDegAsp is therefore not recommended for use in pregnancy at this time [3,23].

## 4. Conclusions

There is a large choice of insulins now available for the treatment of T2D, enabling the clinician to individualise treatment regimens to best suit patient characteristics. Considerations when selecting a treatment schedule for people with T2D include patient preference (for example, for fewer injections or a simpler regimen), variability in meal patterns or daily routine (necessitating a flexible dosing schedule), and a need for better PPG control (by addition of a rapid-acting insulin either as part of a basal-bolus or premixed insulin or IDegAsp treatment regimen).

The IDegAsp co-formulation was developed to provide both FPG and PPG control, with a low risk of hypoglycaemia, from a simple and flexible treatment regimen. Evidence shows that IDegAsp can be used in many situations and is a suitable option for both insulin initiation and intensification. In Australia, where insulin degludec is not available for use, IDegAsp enables patients to still benefit from the ultra-long-acting properties of insulin degludec.

Practical advantages of IDegAsp, compared with premixed or basal-bolus regimens, include straightforward dose titration, greater flexibility in dose timing, reduced injection burden and simplicity of switching, with equivalent glycaemic control, better PPG control, and a lower risk of hypoglycaemia in some circumstances, especially nocturnal hypoglycaemia. IDegAsp is therefore particularly useful for patients who struggle to adhere to complex multidrug regimens or regimens requiring multiple daily injections, those requiring flexibility in the timing of insulin dosing, those failing to achieve glycaemic control despite optimising current non-insulin therapies, or with a PPG spike, with basal or premixed insulins despite successful titration to FPG target, and those at increased risk of hypoglycaemia.

## Figures and Tables

**Table 1:** Key phase 3 clinical trials of IDegAsp in T2D.

Study	Study Design	Mean HbA1c	Mean FPG (mmol/L)	Hypoglycaemia (Overall Confirmed or Nocturnal Confirmed)
**Initiation of IDegAsp (Insulin-Naïve Patients)**				
BOOST JAPAN (Onishi et al.) [45] NCT01272193	Phase 3a 26-week, open-label, treat-to-target *n* = 296 (Japanese) IDegAsp OD vs. glargine U100 OD	ETD IDegAsp/glargine: −0.28% [95% CI −0.46, −0.10]; *p* < 0.01	ETD IDegAsp/glargine: 0.15 [95% CI −0.29, 0.60]; *p* = ns	Overall: ERR IDegAsp/glargine 0.73 [95% CI 0.50, 1.08]; *p* = ns Nocturnal: ERR IDegAsp/glargine 0.75 [95% CI 0.34, 1.64]; *p* = ns
START TWICE DAILY (Franek et al.) [46] NCT01513590	Phase 3b 26-week, open-label, parallel-group, treat-to-target *n* = 394 IDegAsp BID vs. BIAsp 30 BID	ETD IDegAsp/BIAsp 30: 0.02% [95% CI −0.12, 0.17]	ETD IDegAsp/BIAsp 30 BID: −1.00 [95% CI −1.4, −0.6]; *p* < 0.001	Overall: ERR IDegAsp/BIAsp 30: 0.46 [95% CI 0.35, 0.61]; *p* < 0.001 Nocturnal: ERR IDegAsp/BIAsp 30: 0.25 [95% CI 0.16, 0.38]; *p* < 0.001
Kumar et al. PLoS One 2016 [47] NCT01045707 [core] NCT01169766 [ext]	Phase 3 26-week core trial; 26-week extension; open-label, parallel-group, treat-to-target *n* = 530 IDegAsp OD vs. glargine OD	ETD IDegAsp/glargine: −0.08% [95% CI −0.26, 0.09] at week 52	ETD IDegAsp/glargine: 0.28 [95% CI −0.14, 0.69] at week 52	Overall: ERR IDegAsp/glargine: 1.86 [95% CI 1.42, 2.44]; *p* < 0.0001 Nocturnal: ERR IDegAsp/glargine: 0.25 [95% CI 0.14, 0.47]; *p* < 0.0001
SIMPLE USE (Park et al.) [48] NCT01365507	Phase 3b 26-week, open-label, parallel-group, treat-to-target *n* = 276 IDegAsp OD titrated Q2W using simple algorithm vs. IDegAsp OD titrated OW using stepwise algorithm	ETD IDegAspSimple/Stepwise: −0.2% [95% CI −0.4, 0.02]	ETD IDegAspSimple/ Stepwise: −0.4 [95% CI −0.9, 0.09]	Overall: ERR IDegAspSimple/Stepwise: 1.8 [95% CI 1.1, 2.9] Nocturnal: ERR IDegAspSimple/Stepwise: 1.1 [95% CI 0.5, 2.4]
**Intensification from Basal Insulin to IDegAsp**				
Step-by-Step trial (Gupta et al.) [49] NCT02906917	Phase 3 38-week, open-label, treat-to-target *n* = 532 Inadequately controlled on basal insulin ± OADs IDegAsp OD vs. glargine U100 OD + IAsp OD for 26 weeks then IDegAsp OD/BID vs. glargine U100 OD + IAsp OD/BID/TID, for 12 weeks	Weeks 0–26: ETD 0.07% [95% CI −0.06, 0.21] Week 38: ETD 0.09% [95% CI −0.04, 0.22]	Week 26: IDegAsp: −2.3 (SD 2.9) glargine U100 + IAsp: −2.3 (SD 3.3) Week 38: IDegAsp: −2.7 (SD 3.0) glargine U100 + IAsp: −2.3 (SD 3.1)	Overall: IDegAsp/glargine: Weeks 0–26: ERR 0.90 [95% CI 0.67, 1.02] Weeks 0–38: ERR 0.86 [95% CI 0.65, 1.14] Nocturnal: Weeks 0–26: ERR 0.55 [95% CI 0.34, 0.90] Weeks 0–38: ERR 0.61 [95% CI 0.40, 0.93]
Kumar et al. Diabet Med 2017 [50] NCT01045447	Phase 3 26-week, open-label, treat-to-target *n* = 465 IDegAsp OD vs. glargine OD	ETD IDegAsp/glargine: −0.03% [95% CI −0.20, 0.14]; *p* = ns	ETD IDegAsp/glargine: 0.33 [95% CI −0.11, 0.77]; *p* = ns	Overall: ERR IDegAsp/glargine: 1.43 [95% CI 1.07, 1.92]; *p* < 0.05 Nocturnal: ERR IDegAsp/glargine: 0.80 [95% CI 0.49, 1.30]; *p* = ns
Intensify All (Kaneko et al.) [51] NCT01059812	Phase 3 26-week, open-label, treat-to-target *n* = 424 (Asian) Inadequately controlled on basal, premixed or self-mixed (20%–40% rapid-acting) insulin ± metformin IDegAsp BID vs. BIAsp 30 BID	ETD IDegAsp/BIAsp 30: 0.05% [95% CI −0.10, 0.20]	ETD IDegAsp/BIAsp 30: –1.06 [95% CI −1.43, −0.70]; *p* < 0.001	Overall: ERR IDegAsp/BIAsp 30: 1.00 [95% CI 0.76, 1.32]; *p* = ns Nocturnal: ERR IDegAsp/BIAsp 30: 0.67 [95% CI 0.43, 1.06]; *p* = ns
**Intensification from IDegAsp OD to IDegAsp BID**				
Step-by-Step trial (Philis-Tsimikas et al.) [43] NCT02906917	As above	As above	As above	As above
**Switching from Premixed or Basal-bolus Insulin to IDegAsp**				
Intensify Premix I (Fulcher et al.) [41] NCT01009580	Phase 3a 26-week, open-label, treat-to-target *n* = 447 Inadequately controlled with premixed or self-mixed insulin ± OADs IDegAsp BID vs. BIAsp 30 BID	IDegAsp/BIAsp30 ETD: −0.03% [95% CI −0.18, 0.13]	IDegAsp/BIAsp30: ETD: −1.14 [95% CI −1.53, −0.76]; *p* < 0.001	Overall: ERR IDegAsp/BIAsp30: 0.68 [95% CI 0.52, 0.89]; *p* = 0.0049 Nocturnal: ERR IDegAsp/BIAsp30: 0.27 [95% CI 0.18, 0.41]; *p* < 0.0001
Intensify All (Kaneko et al.) [51] NCT01059812	As above	As above	As above	As above
Intensify Premix I/Intensify All pooled analysis (Christiansen et al.) [52] NCT01513590	Pooled analysis of Intensify Premix I and Intensify All Inadequately controlled with premixed insulin ± OADs OR basal or premixed or self-mixed insulin ± metformin, respectively IDegAsp BID vs. BIAsp 30 BID	IDegAsp vs. BIAsp 30: ETD 0.00% [95% CI −0.11, 0.10]; *p* = ns	IDegAsp vs. BIAsp 30: ETD −1.12 [95% CI −1.38, −0.85]; *p* < 0.0001	Overall: ERR IDegAsp vs. BIAsp 30: 0.81 [95% CI 0.67, 0.98]; *p* = 0.03 Nocturnal: ERR IDegAsp vs. BIAsp 30: 0.43 [95% CI 0.31, 0.59]; *p* < 0.0001
Step-by-Step trial (Philis-Tsimikas et al.) [43] NCT02906917	As above	As above	As above	As above
Yang et al. Diab Obesity Metab 2019 [53] NCT02762578	Phase 3a 26-week, open-label, treat-to-target *n* = 543 IDegAsp BID vs. BIAsp 30 BID	IDegAsp vs. BIAsp 30: ETD 0.08% [95% CI −0.20, 0.05]; *p* < 0.0001	IDegAsp vs. BIAsp 30: ETD −1.42 [95% CI −1.74, −1.10]; *p* < 0.0001	Overall: ERR IDegAsp vs. BIAsp30: 0.57 [95% CI 0.42, 0.77]; *p* = 0.0002 Nocturnal: ERR IDegAsp vs. BIAsp30: 0.53 [95% CI 0.33, 0.87]; *p* = 0.0112

BIAsp 30, biphasic insulin aspart 30/70; BID, twice daily; CI, confidence interval; ERR, estimated rate ratio; ETD, estimated treatment difference; IAsp, insulin aspart; IDegAsp, insulin degludec/insulin aspart co-formulation; glargine, insulin glargine; glargine U100, insulin glargine 100 units/mL; ns, not significant; OAD, oral antidiabetic drug; OD, once daily; OW, once weekly; TID, three times daily; T2D, type 2 diabetes; Q2W, every 2 weeks.

## Supplementary Materials

The following are available online at https://www.mdpi.com/2077-0383/9/4/1091/s1, Table S1: Overview of IDegAsp ((Ryzodeg^®^ 70/30) delivery systems [24,56].
