# Supplementary material for: The Clinical Role of Insulin Degludec/Insulin Aspart in Type 2 Diabetes: An Empirical Perspective from Experience in Australia
Source: J Clin Med. 2020 Apr 11;9(4):1091. doi: 10.3390/jcm9041091 (PMC7230791; doi:10.3390/jcm9041091)
Supplement: Supplementary file 1 [file jcm-09-01091-s001.pdf]

Supplementary Materials: The Clinical Role of Insulin Degludec/Insulin Aspart in Type 2 Diabetes: An Empirical Perspective from Experience in Australia

Sarah J. Glastras *, Neale Cohen, Thomas Dover, Gary Kilov, Richard J. MacIsaac, Margaret McGill and Greg R. Fulcher

Table S1. Overview of IDegAsp (Ryzodeg® 70/30) delivery systems [1–3].

	**Ryzodeg® 70/30 FlexTouch®**	**Ryzodeg® 70/30 Penfill® 3 mL**
Description	Ryzodeg 100 units/mL solution for injection in a disposable multidose pre-filled pen (1 to 80U in steps of 1U)<br><br>To be used with NovoFine/NovoTwist injection needles up to 8 mm<br><br>Patients instructed not to use a syringe to remove the solution from the pre-filled pen, to avoid dosing errors	Ryzodeg 100 units/mL solution for injection in a disposable cartridge<br><br>To be used with Novo Nordisk delivery systems (e.g. NovoPen®) and NovoFine/NovoTwist injection needles up to 8 mm<br><br>Patients instructed not use the insulin level indicator on the insulin delivery system to measure dose
How to use	Subcutaneous injection into abdomen, upper arms or the front of the thighs (rotating injection sites)<br>• Pinch the skin between two fingers, push the needle into the raised skin, and inject the full dose of insulin under the skin<br>• Keep the needle under the skin for at least six seconds with the dose button fully depressed until the needle has been withdrawn from the skin<br>• Apply gentle pressure over the injection site for several seconds<br>Patient recommendations<br>• Always use a new needle for each injection to prevent contamination<br>• Do not use Ryzodeg® 70/30 in insulin infusion pumps<br>• Always carry a spare FlexTouch®/Penfill® in case your in-use FlexTouch®/Penfill® is lost or damaged	
When to use	Once- or twice-daily with main meal(s)<br>Recommended total daily starting dose for T2D in insulin naïve patients: 10U, followed by individual adjustments	
Monitoring	Patients advised to<br>• measure plasma glucose regularly<br>• tell their doctor if they often have hypoglycaemia, trouble recognising hypoglycaemia, trouble with their eyesight, or if they become pregnant<br>Close glucose monitoring recommended<br>• on transferring from other insulin medicinal products<br>• in the elderly or in renal and hepatic impaired patients	

References

1. Ryzodeg® 70/30 FlexTouch®/Penfill® PI. Availabe online: http://www.novonordisk.com.au/content/dam/australia/affiliate/www-novonordisk-au/Health%20Care%20Professionals/Documents/Ryzodeg%2070-30_pi1.pdf (accessed on 2 December, 2019).

2. Ryzodeg® 70/30 FlexTouch®. Consumer Medicine Information. Availabe online: http://www.novonordisk.com.au/content/dam/australia/affiliate/www-novonordisk-au/Health%20Care%20Professionals/Documents/Ryzodeg%2070-30%20FlexTouch_cmi1.pdf (accessed on 2 December, 2019).
3. Ryzodeg® 70/30 Penfill®. Consumer Medicine Information. Availabe online: http://www.novonordisk.com.au/content/dam/australia/affiliate/www-novonordisk-au/Health%20Care%20Professionals/Documents/Ryzodeg%2070-30%20Penfill_cmi1.pdf (accessed on 2 December, 2019).
